# Higher Medical Education—State Boards of Medical Examiners

**Published:** 1891-04

**Authors:** 


### HOMŒOPATHIC PHYSICIAN,

A MONTHLY JOURNAL OF

HOMŒOPATHIC MATERIA MEDICA AND CLINICAL MEDICINE.

"If our school ever gives up the strict inductive method of Hahnemann, we are lost, and deserve only to be mentioned as a caricature in the history of medicine."—CONSTANTINE HERING.

Vol. XI. APRIL, 1891. No. 4.

### EDITORIAL.

"HIGHER MEDICAL EDUCATION." "STATE BOARDS OF MEDICAL EXAMINERS."—For some time past our esteemed allopathic contemporaries have been urging the necessity of higher medical education and the need of boards of medical examiners. To what end? Ostensibly that the public may be benefited by having in the medical profession only those best fitted for the practice of medicine; at the same time they trust to be able to work harm to Homœopathy.

That the highest medical education is desirable, we do not question, but the highest is of no value unless it be the right—unless it be of a character to enable its recipients to do better for the sick than allopathy has done and is doing.

That the most advanced education in allopathy is as much of a failure now as it has always been is illustrated in almost every journal published by that school. After reading articles written by the leading lights on the treatment of various diseases, we always wonder how the patients of the second-rate men ever survive, and we can only conclude that there are many who recover in spite of the treatment.

When we read their statistics, and compare the great mortality with what we know of the results of genuine homœopathic treatment, we can only stand aghast and ask how much longer is this slaughter to continue, even though it be done in the name of so-called scientific medicine?

Homœopathicians can show that many deaths are the immediate result of too much drugging, and that many living deaths, which are attended by much suffering, are due to the same cause. We do not hesitate to affirm that allopathic treatment is responsible for more suffering than any other one cause, not even excluding rum.

If higher medical education would only teach the allopathists this fact: that drugs kill more people than disease, there would be an advance greater than they have ever made. We have little hope of this, however, whenever we take up any of their journals in which are related cases of disease with treatment.

In a recent number of the London *Lancet* we read of the case of a woman who was under the treatment of a leading physician in London (higher medical education is supposed to prevail in that town), and if a fourth-rate homœopathician could not have done better we should blush for Homœopathy and declare it a fraud. The case was one of facial neuralgia, which did not yield to drugging. The pain was at first limited to the right inferior dental nerve, and that was stretched and then divided. This was for a time "successful" (?), but after a time the pain returned with greater violence. Another portion of the nerve was removed. The pain again recurred, this time extending along the course of the gustatory nerve. A portion of the dental and gustatory nerves was divided in the pterygoid region. "This failed to give relief, and very severe pains were experienced in the alveolar process of the upper jaw of the same side." It was then determined to remove the Gasserian ganglion. This was done, and there followed suppuration of the eyeball and it was necessary to remove it. And still the original pain continued!

What homœopathician who, after reading this, but would exclaim, "How barbarous"?

We should expect better results from an ignorant layman with the poorest work on homœopathic domestic practice.

And yet this was done in London, by a leading physician, who had all the advantages of higher medical education!

Although such a performance as this is not common, there is not wanting other evidence to show what higher medical education is doing for allopathy—and undoing for its victims.

In a more recent number of the *Lancet* is an analysis of 1,000 cases of pneumonia treated in the London Hospital. In 285 cases there was a mortality of 33.9 per cent.

"Two *great* (we italicize) methods of treatment have been examined in detail as to their influence upon the mortality from the disease. [The mortality was from the treatment.] In the one we find that the chief remedies were aimed at relieving the condition of the lung; and while stimulants were freely administered with the object of whipping up the flagging heart, the cause of its physical lameness was allowed to proceed unmolested. [The mortality would have been much lessened if the entire organism had been unmolested.] The result was that among 552 cases so treated the mortality exceeded 23 per cent., although alcohol was exhibited in no less than 70 per cent. In 108 cases of similar severity to the foregoing the treatment consisted in the systematic reduction of temperature by means of sponging or ice-cradling. Of this number only 45 (41 per cent.) received alcohol, and only 10 per cent. died."

Is there any treatment that can show more impotence for good and more potence for harm?

No treatment would give better results.

If Malthus had known the results of allopathic treatment, we are sure he would never have thought the earth could be overpopulated.

If Hahnemannian Homœopathy could not do vastly better than the highest allopathic medical education enables its followers to do, we should denounce it as false, and cast it aside as of no value. G. H. C.

---

That allopathic treatment is not the only method of killing, the following will show:

An example of what is done in the name of Homœopathy may be found in the New York *Medical Times* for February

The same case is reported in the *Hahnemannian Monthly* for February. The one who first treated the case calls himself a homœopath, and those who assisted are also known by that name. We find from the two articles that a young lady, æt. nineteen years, had suffered for several years with naso-pharyngeal catarrh and otitis media. On October 3d the first man was called, and "found her complaining of lassitude, headache, and intense pain in left ear, which was *discharging profusely*" (we italicize). "The ears were loosely packed with Boric acid, which was removed every night by syringing. On the seventh day after my first visit [*the discharge*, according to the account in the *Hahnemannian Monthly, ceased on that day*] she suddenly developed tonic and clonic convulsions. * * * Up to this time the treatment consisted in the application of heat to the head, Mustard over the mastoid and nape of the neck, and Belladonna, Hepar-sulph., Acetanilide, and Codeine internally."

Consultation was then called, an incision was made over the mastoid "with negative results." Then, "the next morning Dr. —— trephined the mastoid, but without any indication of pus or diseased bone. It was then decided to explore the brain itself, * * * but incision of the dura and pia mater and probing in every direction failed to discover pus. The patient rallied nicely from the operation, and for several hours was apparently relieved, but delirium and restlessness again recurring, she was given Phenacetine and Morphia with good results. On the following day she was rational, took bovinine and chicken broth, but at eight P. M. died suddenly." The writer in the *Hahnemannian* assures us death was not hastened by the operation.

Post-mortem showed no evidence of mastoid disease, but there was pus on the upper surface of the cerebellum.

It will be noted that the convulsions did not appear until the profuse discharge from the ears had been suppressed. It will also be seen that the patient was not treated homœopathically from the beginning, and every homœopathician will also note that the cause of death was the treatment given in the name of Homœopathy.

We feel that language is not sufficient to fitly characterize the action of these men who so bunglingly managed the case. And what they did was as followers of Hahnemann! What should be said of and done to such as these who dare drag into the mire the fair name of Hahnemann and his honest followers?

If the poor victim had been related to us in any way, we should have demanded the services of the coroner, and thus placed the cause of death where it rightly belongs—to the infernal treatment given.

And yet we are asked to drop the term "mongrel." We are deterred from using a harsher and more expressive term only because politeness to our readers forbids. We should like to see the men who treated this case given the same treatment. Then, possibly, they might be brought to a realization of what they have done. G. H. C.

